# Non-invasive Positive Pressure Ventilation for Acute Cardiogenic Pulmonary Edema and Chronic Obstructive Pulmonary Disease in Prehospital and Emergency Settings

**DOI:** 10.7759/cureus.15624

**Published:** 2021-06-13

**Authors:** Ansha P Abubacker, Andrew Ndakotsu, Harsh V Chawla, Aimen Iqbal, Amit Grewal, Revathi Myneni, Govinathan Vivekanandan, Safeera Khan

**Affiliations:** 1 Emergency Medicine, California Institute of Behavioral Neurosciences & Psychology, Fairfield, USA; 2 Internal Medicine, California Institute of Behavioral Neurosciences & Psychology, Fairfield, USA

**Keywords:** chronic obstructive pulmonary disease, pulmonary edema, non-invasive ventilation, heart failure, cpap, bipap, pulmonary diseases

## Abstract

Non-invasive ventilation is an important intervention in treating acute respiratory failure caused by acute cardiogenic pulmonary edema (ACPE) and acute exacerbations of chronic obstructive pulmonary disease (COPD). Although there are studies that give evidence on the efficacy and safety of non-invasive ventilation over standard medical care for COPD and cardiogenic pulmonary edema, less are known about the form of non-invasive ventilation, continuous positive airway pressure (CPAP), or bilevel positive airway pressure (BiPAP) as an effective intervention for respiratory failure and its efficacy and safety in prehospital settings. We conducted a systematic review by using PubMed and Google Scholar as databases for collecting studies related to the effectiveness of CPAP and BiPAP for cardiogenic pulmonary edema and COPD; the major outcome studied was reducing rates of endotracheal intubation secondary and tertiary outcomes included mortality reduction and shortening length of hospital stay. The study follows the guidelines of the Preferred Reporting Items for Systematic Review and Meta-Analysis (PRISMA) checklist 2009. Sixteen studies were identified, including systematic reviews, randomized control trials, and observational studies. Studies published on or after 2010 in a population greater than 40 years old suffering from acute COPD and cardiogenic pulmonary edema were taken for review. Studies that described other respiratory diseases treated with non-invasive ventilation were excluded. Quality appraisal was done using the Cochrane risk bias tool for randomized control trials, Amstar-2 for systematic reviews, and New Castle Ottawa Tool for observational studies. Five studies compared the effectiveness of CPAP and BiPAP with standard medical care in prehospital and emergency settings. Six studies described prehospital intervention. Both forms of non-invasive ventilation were equally significant and effective. Prehospital use had tremendously reduced intubation rates, with not much variability noticed for mortality and hospital stay. Non-invasive ventilation is an effective measure for respiratory failure secondary to COPD and ACPE. Early out of hospital utilization of CPAP and BiPAP reduces the rate of invasive ventilation and reduces complications due to endotracheal intubation. Endotracheal intubation is associated with a considerable incidence of complications like failed intubation, hypotension, or circulatory arrest, even if the emergency physician is well trained, making these forms of non-invasive ventilation safe and effective interventions in the prehospital settings.

## Introduction and background

Respiratory failure due to acute exacerbations of chronic obstructive pulmonary disease (COPD) and acute cardiogenic pulmonary edema (ACPE) caused by left ventricular failure are common presentations encountered in prehospital and emergency care settings [1]. COPD, according to World Health Organization, is the fifth global burden in public healthcare [2]. Cardiogenic pulmonary edema is another cause of hypoxemic respiratory failure commonly treated with medications like morphine, nitroglycerine, oxygen therapy, and if failed with endotracheal intubation [3]. The mode of airway management and application of airway supporting devices are still a prior topic in research studies for acute respiratory failures [3].

A mode of ventilatory support in which positive pressure is delivered into the lungs without an invasive endotracheal airway is called non-invasive ventilation [4]. It is frequently used to support patients with acute respiratory failure. Assessing a patient for non-invasive ventilation includes identifying the conditions responsible for acute respiratory failure typically responsive to non-invasive ventilation. The major benefit of non invasive ventilation is to decrease intubation rates and related complications. The trials with noninvasive ventilation should be kept short if there is no clinical improvement as the delayed mechanical ventilation can cause poor outcome. The contraindication to noninvasive ventilation includes need for emergent intubation. Absolute contraindications include cardiac or respiratory arrest, severe respiratory distress, unstable cardiac arrythmias and relative contraindications include hemodynamic instability, facial abnormalities, severe airway obstructions or in conditions with inability to protect airways.

The past few decades have witnessed the use of non-invasive ventilation in substantially treating acute respiratory failure secondary to acute exacerbation of COPD and ACPE in emergency settings along with standard medical care [5]. Even though there are pieces of evidence supporting the use of non-invasive ventilation for treating these conditions, there are few systematic reviews written on safety and efficacy on comparing the different forms of non-invasive ventilation and use of its early intervention in prehospital settings helping to reduce morbidity and mortality of patients by reducing the need for invasive intubation, thereby reducing complications like ventilator-associated pneumonia [3].

An observational study in France in 2013 has noted reduced intubation rates with no significant risk reduction in mortality and hospital stay [6]. Prehospital non-invasive ventilation as a primary intervention for COPD and ACPE by certain studies found no significance in mortality and hospital stay other than reducing endotracheal intubation rates [3,7,8].

The study focuses on answering questions like comparing the efficacy and safety of continuous positive airway pressure (CPAP) with bilevel positive airway pressure (BiPAP) in treating acute exacerbation of COPD and cardiogenic pulmonary edema and evaluating the effects of the early prehospital intervention of CPAP in treating COPD and cardiogenic pulmonary edema in reducing invasive intubation and reducing mortality. acute respiratory failure due to ACPE and COPD were taken for review as they are commonly encountered in emergency settings and there is an increasing trend towards using non invasive ventilation as a first line treatment for acute exacerbations of COPD and ACPE in emergency settings, hence there is a need for assessing efficacy of non-invasive ventilation for decreasing mortality rate, intubation rate and length of hospital stay and use of an early intervention in prehospital settings for these diseases in particular in more upcoming studies.

A systematic review based on this topic may help solve this issue by knowing about the right form and time of non-invasive ventilation for acute hypercapnic respiratory failure. Hence, a systematic review is conducted using PubMed and Google Scholar as databases that included observational studies, randomized control trials, and systematic reviews on this topic from 2010.

## Review

Method

Aim

Our study aimed at evaluating the effects of different forms of non-invasive ventilation CPAP and BiPAP for COPD and cardiogenic pulmonary edema. We also aimed to study the efficacy and safety of prehospital intervention with non-invasive ventilation.

Information Sources

We identified relevant studies that dealt with the need for non-invasive ventilation as an early intervention for exacerbations of COPD and cardiogenic pulmonary edema in prehospital and emergency settings. Our studies included randomized control trials, systematic reviews done on randomized and non-randomized control trials, and observational studies published in journals from 2010. A thorough search was done using PubMed and Google Scholar as databases from inception to February 8, 2021.

**Figure 1:**
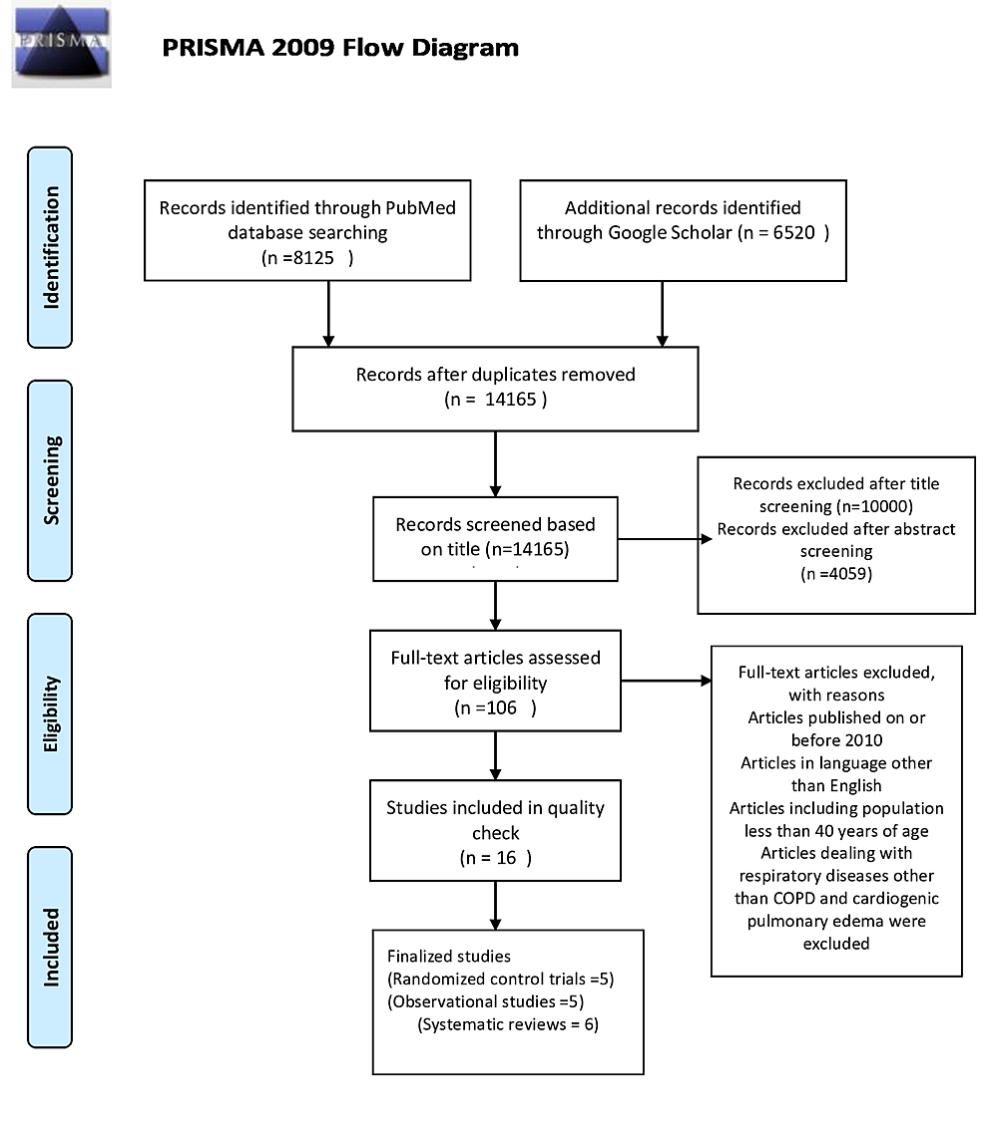
PRISMA 2009 Flow Diagram PRISMA - Preferred Reporting Items for Systematic Reviews and Meta-Analysis

The whole study is conducted abiding by the rules of the Preferred Reporting Items for Systematic Review and Meta-Analysis (PRISMA) 2009 Guidelines [9]. A complete PRISMA flow diagram is shown in Figure 1 [9].

Search Strategy

Appropriate medical subject headings (MeSH) were identified for keywords and the MeSH strategy was developed for PubMed search by using Boolean AND and OR. The databases and search strategy developed for searching relevant articles are shown in Table 1.

**Table 1:** Databases and Search Strategy

Databases	Search Strategy	Search Results
Google Scholar Keywords	COPD and Non-Invasive Ventilation and CPAP and BiPAP and Pulmonary Edema and Heart Failure and Pulmonary Disease	6520
PubMed Mesh Strategy	(( "Continuous Positive Airway Pressure/adverse effects"[Majr] OR "Continuous Positive Airway Pressure/trends"[Majr] )) OR ( "Continuous Positive Airway Pressure/adverse effects"[Mesh:NoExp] OR "Continuous Positive Airway Pressure/trends"[Mesh:NoExp] ) OR (( "Noninvasive Ventilation/adverse effects"[Majr] OR "Noninvasive Ventilation/trends"[Majr] )) OR ( "Noninvasive Ventilation/adverse effects"[Mesh:NoExp] OR "Noninvasive Ventilation/trends"[Mesh:NoExp] ) AND ("Respiratory Insufficiency/therapy"[Majr]) OR "Respiratory Insufficiency/therapy"[Mesh:NoExp] OR ("Pulmonary Disease, Chronic Obstructive/therapy"[Majr]) OR "Pulmonary Disease, Chronic Obstructive/therapy"[Mesh:NoExp] AND (( "Heart Failure/complications"[Majr] OR "Heart Failure/therapy"[Majr] )) OR ( "Heart Failure/complications"[Mesh:NoExp] OR "Heart Failure/therapy"[Mesh:NoExp] ) OR ("Pulmonary Edema/therapy"[Majr]) AND "Pulmonary Edema/therapy"[Mesh:NoExp] OR Noninvasive ventilation OR CPAP AND Respiratory failure OR COPD AND Heart failure OR cardiogenic pulmonary edema	8125

Data Collection

Identified articles by search strategy were transported to endnote, and duplicates were removed. Two authors first screened articles independently with titles and abstracts. Articles that gave data other than the effect of non-invasive ventilation in COPD and pulmonary edema were removed without any discrepancies. Articles were screened for eligibility criteria by another set of two independent authors.

Eligibility Criteria

Papers published in or after 2010 in English language papers with full text extracted were taken for review. Adults of age greater than 40 years and patients without the need for immediate intubation with no contraindications for non-invasive ventilation were included. Studies that dealt with non-invasive ventilation CPAP and BiPAP for COPD, cardiogenic pulmonary edema, and prehospital non-invasive ventilation were collected for data extraction.

Data Extraction

Data were extracted by two investigators from studies that met the inclusion criteria. The data were collected on a form that included study design, the number of patients reported outcomes that included endotracheal intubation rates, mortality rates, and length of hospital stay.

Study Quality

The quality of studies was assessed using the Cochrane risk bias tool for randomized control trials, the Amstar-2 checklist for systematic reviews, and the New Castle Ottawa tool for observational studies. Studies with low quality were excluded from the review.

Result

After an extensive search, 8,125 studies from PubMed and 6,520 studies from Google Scholar were located, making a total of 14,645 articles. A total of 480 duplicates were found and removed using the endnote basic online version, making 14,165 articles for screening. All the 14,165 articles were screened with their titles and abstracts by two independent authors. Around 10,000 articles after title screening and 4,059 articles after abstract screening were excluded. Articles that dealt with the efficacy of non-invasive ventilation for COPD and cardiogenic pulmonary edema over standard medical therapy and prehospital intervention of non-invasive ventilation were selected for further review. Around 106 articles in this way were identified. Obtained potentially relevant articles were evaluated for eligibility based on inclusion and exclusion criteria. Eighteen articles published before 2010 were excluded. Eighty-eight articles were further screened for eligibility criteria. Papers published in a language other than English dealt with the intervention of non-invasive ventilation for respiratory failure other than COPD and cardiogenic pulmonary edema were excluded. Some articles were excluded as they were dealing with domiciliary care with non-invasive ventilation for chronic respiratory failure. Articles comparing non-invasive ventilation with invasive ventilation removed and studies comparing non-invasive ventilation with standard medical therapy were only included. Articles that studied pressure support and pressure-controlled CPAP were excluded, and studies were limited to comparing CPAP with BiPAP.

 Sixteen moderate to high-quality studies were taken for the systematic review. The summary of the studies included in the review is shown in Table 2.

 

**Table 2:** Summary of studies taken for systematic review NIPPV - Non-invasive Positive Pressure Ventilation, NIV - Non-invasive Ventilation, ACPE - Acute Cardiogenic Pulmonary Edema, CPAP - Continuous Positive Airway Pressure, BiPAP - Bilevel Positive Airway Pressure, RCT - Randomized Control Trial, ICU - Intensive Care Unit,  COPD - Chronic Obstructive Pulmonary Disease, SR - Systematic Review, MA - MetaAnalysis, ARF - Acute Respiratory Failure

Author/Year of Publication	Study Type	Intervention	No of Patients	Results	Conclusion
Vital et al. 2013 [10]	SR&MA	CPAP and BiPAP in cardiogenic pulmonary edema	2,916	NIPPV reduced mortality, intubation rates, and length of hospital stay in patients with cardiogenic pulmonary edema	No variation observed in CPAP and BiPAP
Osadnik et al. 2017 [11]	SR&MA	NIV for COPD	1,264 Mean age 66.8 years	NIPPV reduced mortality, intubation rates, and length of hospital stay in patients with COPD	NIV is effective in COPD
Mccurdy et al. 2012 [12]	SR&MA	Evidence-based analysis for NIV and COPD	1,000	Endotracheal intubation rates, hospital stay, and mortality were reduced in NIV intervention on COPD patients	NIV is effective as a first-line intervention in acute exacerbations of COPD
Mariani et al. 2011 [13]	SR&MA	CPAP vs. NIPPV vs. oxygen in cardiogenic pulmonary edema	3,041	CPAP and NIPPV reduced mortality and intubation rates in cardiogenic pulmonary edema	No difference between CPAP and NIPPV
Berbentz et al. 2019 [14]	SR	CPAP or BiPAP for cardiogenic pulmonary edema	2,664	NIPPV reduces hospital mortality intubation rates. There is probably little difference in acute myocardial infarction incidence with NIPPV	NIPPV is a safe and effective intervention for ACPE
Bakke et al. 2014 [3]	SR	CPAP as a prehospital intervention for acute respiratory failure	2,092	Reduction in intubation rates noted	The current evidence shows no difference in mortality or hospital length of stay, but a trend towards reduction of intubation rates noted
Roosler et al. 2012 [7]	RCT	Need for out of hospital intervention with NIV in acute respiratory failure	51 - 26 with standard medical therapy 25 with NIV	Six patients with standard medical therapy needed invasive intubation. Only one patient in NIV needed intubation	OOH NIV is safe and effective in acute respiratory failure with standard medical therapy
Fontin et al. 2011 [15]	RCT	CPAP for cardiogenic pulmonary edema	124	Patients received prehospital and ICU care CPAP. Death occurred in the statistically same figures	No significant difference noted for usual medical therapy and CPAP intervention in prehospital or ICU setting
Ferrari et al. 2010 [16]	RCT	Comparing CPAP with NIPPV for acute cardiogenic pulmonary edema	Out of 80 patients 40 received NIPPV, and another 40 received CPAP	No patients needed endotracheal intubation in CPAP intervention three patients required in NIPPV Intervention	No significant difference in mortality. No significant difference in endotracheal intubation. CPAP is more effective as it is cost-effective and more convenient to use
Ducros et al. 2011 [8]	RCT	CPAP for cardiogenic pulmonary edema	207	CPAP 60 min prehospital 120 min ICU has made a good tolerance in patients	CPAP effective intervention for cardiogenic pulmonary edema
Belenger et al. 2017 [17]	RCT	NIV vs. CPAP for acute respiratory failure	110 - 56 with NIV 54 received CPAP	Both reduced length of hospital stays morality and intubation rates	NIV and CPAP had no significant difference
Willimore et al. 2015 [18]	Before and after observational study Ottawa	Prehospital positive pressure ventilation	341 patients Mean age 71.5 ACPE 18.9 COPD 21.9	The overall effect in mortality is greater in the after-group	No improvement in morbidity, mortality, and hospital stays
Luiz et al. 2016 [19]	Observational study Germany	CPAP for cardiogenic pulmonary edema and COPD in emergency medicine department	57 patients 35 with ACPE 22 with COPD	Seven patients required secondary intubation in COPD. Six patients are required in ACPE.	CPAP is an effective measure in COPD and ACPE for reducing the rate of intubation
Contou et al. 2013 [6]	Observational cohort study at French University Hospital	A nurse-driven cohort study for effectiveness of NIV	242	Endotracheal intubation rates reduced to 15%, with a mortality rate of only 5%	NIV effective measure for ARF
Pirrachio et al. 2013 [20]	Observational study	CPAP in cardiogenic pulmonary edema	2,986	CPAP intervention reduced intubation rates	CPAP effective treatment for cardiogenic pulmonary edema
Aliberti et al. 2018 [21]	A multicenter prospective observational study in Italy in 22 ED	Non-invasive ventilation in acute cardiogenic pulmonary edema	1,293	ARF treated as follows CPAP for 788, BiPAP for 232, oxygen therapy for 273 patient’s 3% in each intervention had early mortality, but treatment failure was halved with NIV interventions compared to oxygen alone	NIV seems to be the first choice of treatment of ARF due to ACPE

Discussion

Our study shows that there is a tremendous decrease in intubation rates and mortality rates on early intervention with CPAP and BiPAP with no significant reduction in length of hospital stay. Both CPAP and BiPAP were equally effective in managing acute respiratory failure.

CPAP v/s BiPAP for Reducing Intubation Rates, Mortality, and Length of Hospital Stay

Five studies out of 16 studies collected for review were comparing the effects of different forms of non-invasive ventilation [10,13,16,17,21]. Outcomes measured included rate of endotracheal intubation, mortality rate, length of hospital stay in patients suffering from acute respiratory failure from COPD and cardiogenic pulmonary edema.

Two systematic reviews [11,14], two randomized control trials [16,17], and one observational study [21] were reviewed for comparison. Although the studies conducted earlier suggest better efficacy on patients with COPD and ACPE on intervention with non-invasive ventilation over standard medical care, fewer studies compared the effect of different forms of non-invasive ventilation on these patients. The studies that compared CPAP and BiPAP for either COPD or cardiogenic pulmonary edema were taken into account for review, which gave the outcomes we studied.

In a systematic review and meta-analysis of randomized control trials conducted by Mariani et al. on non-invasive ventilation for cardiogenic pulmonary edema, a total of 3,041 ACPE patients randomized were 1,044 assigned to BiPAP; 1,160 to CPAP; and 837 for standard medical therapy [13]. BiPAP was associated with a 20% reduction in mortality. No significant differences between BiPAP and CPAP on mortality were noted, 57% reduction in intubation rates was found with CPAP, and 52% with NIPPV. No significant difference was noted between the two ventilatory modes. No effect on myocardial infarction risk was noted in both ventilator modes. No significant difference was noted with the length of hospital stay. The physiological benefit of BiPAP was an improvement in oxygen saturation in patients compared to CPAP. Another systematic review by Vital et al. [10] included 32 studies for review that had concluded a reduction in mortality and intubation rate with no difference in hospital length of stay.

A prospective randomized control trial was conducted by Belenguer et al. [17] on the same comparison of BiPAP (n=56) and CPAP (n=54) in cardiogenic pulmonary edema and a similar randomized control trial by Ferrari et al. [16] on 80 patients with 40 on CPAP and 40 on BiPAP had found out similar outcomes. Both interventions had similar intubation rates and a similar reduction in mortality and hospital stay. This study also observed an improved partial pressure of oxygen with BiPAP compared to CPAP. A real-life multicenter observational study was done on patients with cardiogenic pulmonary edema by Aliberti et al. [21] in 22 emergency departments in Italy with CPAP and BiPAP and oxygen interventions for ACPE. Out of the two percent, the population that required endotracheal intubation more was with BiPAP compared with oxygen intervention and CPAP. But the failure rate was about 33% with oxygen-only intervention and the greatest success rate with CPAP. Comparing CPAP and BiPAP given an equal reduction in mortality rate compared to standard medical therapy.

**Figure 2:**
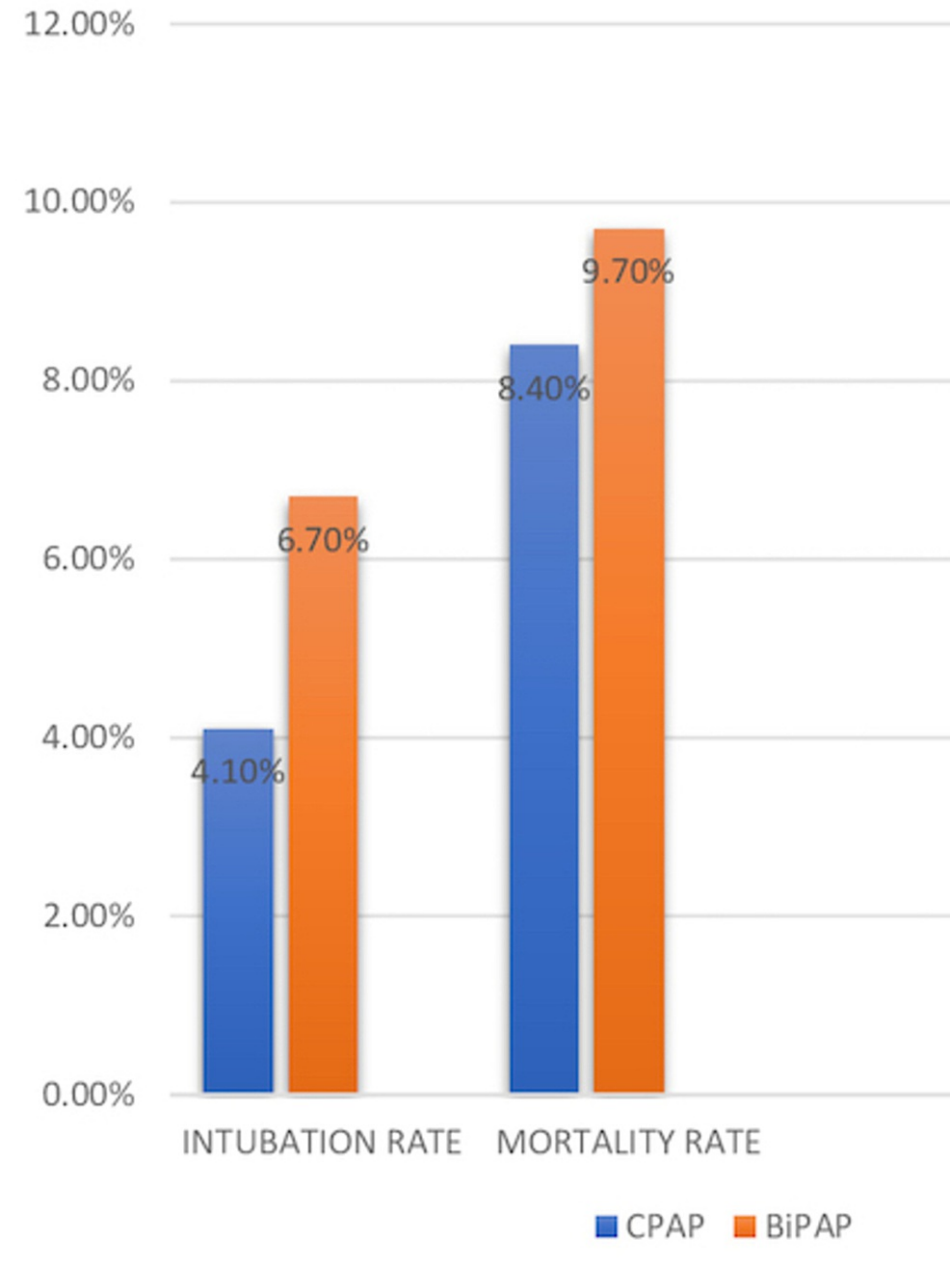
Rates of mortality and intubation with CPAP and BiPAP CPAP - Continuous Positive Airway Pressure, BiPAP - Bilevel Positive Airway Pressure

In this five studies [10,13,16,17,21], a total of 1,984 patients received CPAP and 1,419 received BiPAP. The mortality rate with CPAP was 168 out of 1,984 and that with BiPAP was 139 out of 1,419. It comprised 8.4% of the population with CPAP and 9.7% of the population who received BiPAP. Intubation rates with CPAP and BiPAP were 83 out of 1984 and 96 out of 1419 respectively. It means 4.1% of the population with CPAP and 6.7% with BiPAP needed endotracheal intubation. Length of hospital stay was not affected by the kind of intervention used but was found to be reduced than treatment with standard medical care alone. For better understanding, a graphical representation of a comparison of CPAP and BiPAP for acute respiratory failure caused by COPD and cardiogenic pulmonary edema is shown in Figure 2.

Prehospital CPAP: A Remedial Measure to Reduce Mortality and Intubation Rates in COPD and Acute Cardiogenic Pulmonary Edema

Prehospital non-invasive ventilation can be effective in treating acute exacerbation of COPD and cardiogenic pulmonary edema. Six studies [3,7,8,15,18,19] were reviewed for the effectiveness of prehospital or out-of-hospital use of CPAP for reducing mortality intubation rates and hospital stay length. Reducing endotracheal intubation rate in other ways helps in reducing the risk of ventilator-associated pneumonia, length of hospital stay, and mortality.

One systematic review [3], three randomized control trials [7,8,15], and two observational studies [18,19] were taken into account for review. A systematic review done by Bakke et al. [3] showed a great decrease in intubation rates when CPAP is given in prehospital settings but no reduction in mortality and length of hospital stays. Willimore et al. [18] had conducted a before and after study at Ottawa regarding prehospital CPAP effectiveness for ACPE and COPD. They found out no significant role in reducing mortality and morbidity in patients after intervention. The following study concluded a decreased effectiveness of prehospital CPAP over usual standard medical care. Another observational study conducted by Luiz et al. [19] on intervention with prehospital CPAP with 35 ACPE and 22 COPD patients at an oxygen flow of 21.8+/_5.8L/min and PEEP of 6.1+/_1.6 bar. CPAP was found to reduce mortality, hospital stay, and intubation rates effectively. Intubation rates were more in patients with ACPE complicated with acute coronary syndrome compared to patients with cardiogenic pulmonary edema alone. Fontin et al. have done a randomized control trial on CPAP for prehospital use in patients with cardiogenic pulmonary edema, 16 patients were given CPAP, and 62 patients with usual medical care were analyzed and compared for the benefits of CPAP over standard medical care, even though endotracheal intubation rates were reduced, no reduction in morbidity and mortality occurred [15].

Despite the benefits of CPAP for cardiogenic pulmonary edema, the study adheres to a treatment protocol with low dose morphine, furosemide, and oxygen for ACPE because of limited evidence of prehospital CPAP improving morbidity and mortality rate. Roosler et al. conducted a randomized trial that included prehospital non-invasive ventilation for all kinds of respiratory failure, including cardiogenic pulmonary edema and COPD [7]. They suggested out-of-hospital non-invasive ventilation as a feasible option for treating COPD and ACPE and should be considered a first-line treatment option for any respiratory failure. One observational study even recommended using CPAP prehospital setting even when the distance to the hospital is short. A randomized control trial by Ducros et al. had concluded the benefits of early prehospital intervention with CPAP in cardiogenic pulmonary edema, including improvement of left ventricular ejection fraction [8].

**Figure 3:**
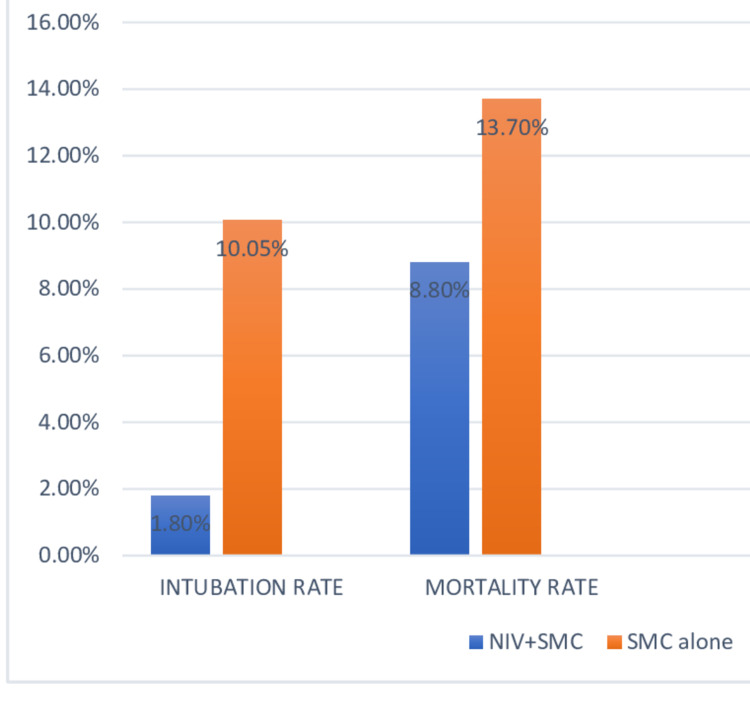
Rates of intubation and mortality of prehospital CPAP compared with standard medical care alone NIV - Non-invasive Ventilation, SMC - Standard Medical Care, CPAP - Continuous Positive Airway Pressure

A total of 1,085 patients received prehospital non-invasive ventilation along with standard medical care and 1,174 patients received standard medical care alone. The intubation rate with NIV was only 1.8% compared to 10.05% for standard medical care alone. Mortality rates were almost halved on intervention with prehospital NIV along with standard medical care. A graphical representation of a comparison of standard medical combined with non-invasive ventilation and non-invasive ventilation alone is demonstrated in Figure 3.

Non-invasive Ventilation for ACPE and COPD in Emergency Settings

**Figure 4:**
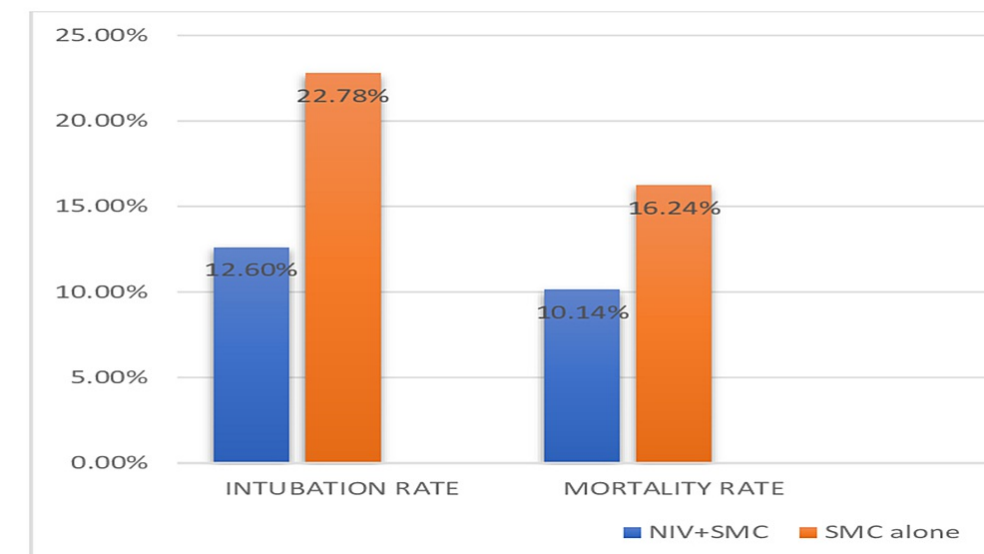
Rates of mortality and intubation with non-invasive ventilation compared to standard medical care alone NIV - Non-invasive Ventilation, SMC - Standard Medical Care

Among the studies reviewed about the importance of giving non-invasive ventilation to patients suffering from acute exacerbation of cardiogenic pulmonary edema and COPD. Six of the studies compared different forms of non-invasive ventilation, including CPAP and BiPAP in emergency settings [3,6,11,12,14,20]. These studies had given the significance of the use of CPAP for reducing endotracheal intubation rates and mortality in COPD and ACPE patients. Intubation rates and mortality rates were reduced to half on intervention with non-invasive ventilation along with standard medical care compared to standard medical care alone. The data obtained are pictorially represented in a bar graph for better understanding in Figure 4.

Limitations

We included only papers published in the English language with publication after 2010, so we might have missed some relevant papers; we noticed some German, French, and Spanish papers without English translation. During the discussion on CPAP comparisons with BiPAP for efficacy in treating COPD and cardiogenic pulmonary edema, not many studies falling under inclusion criteria for comparison in CPAP were found. So, the reliability of that comparison may apply only to cardiogenic pulmonary edema, and intervention with BiPAP may be more effective for cardiogenic pulmonary edema. Most RCT and systematic reviews on RCT included non-blinded trials, so there is a possibility of bias. We have included observational studies from Italy, Ottawa, France, and Germany. Considering medical facilities and patient tolerance in different locations to be different, there can be a bias according to this in our study.

Future Research Question to Be Answered

The following study was conducted by including participants from different reviews with different comorbidities and of different age groups, focusing on certain age group, and with same comorbidities in future researches can let us know on the type of people more benefited with the interventions studied. Although the prehospital intervention had some positive results on reducing intubations, cost-effectiveness and safety on the following are still questions to be answered.

## Conclusions

We found that non-invasive ventilation in the form of CPAP and BiPAP effectively reduces intubation rates, hospital mortality, and length of hospital stay. No significant difference is noted between the two interventions. Early prehospital use of CPAP and BiPAP can be considered to reduce intubation rates. From our study, we found it to be effective in reducing mortality with no significance in the length of hospital stay. Based on our research CPAP or BiPAP is found to be significantly effective in emergency settings with fewer failure rates and should be used as a first-line practice for any acute case of COPD and cardiogenic pulmonary edema.
